# COVID-19 pandemic-induced physical inactivity: the necessity of updating the Global Action Plan on Physical Activity 2018-2030

**DOI:** 10.1186/s12199-021-00955-z

**Published:** 2021-03-07

**Authors:** H. Amini, S. Habibi, A. H. Islamoglu, E. Isanejad, C. Uz, H. Daniyari

**Affiliations:** 1 Department of Physical Education & Sport Sciences, Tolou-e-Mehr Non-profit Institute of Higher Education, Qom, Iran; 2 Physical Education Faculty, Khorasgan Branch, Islamic Azad University, Isfahan, Iran; 3 Nutrition and Dietetics Department, Faculty of Health Sciences, Marmara University, Istanbul, Turkey; 4 Department of Education Area of Qom, Qom, Iran; 5 Physical Medicine and Rehabilitation Clinic, Kirikkale High Specialized Hospital, Kirikkale, Turkey

**Keywords:** COVID-19 pandemic, Physical activity, Global action plan

## Abstract

To prevent and reduce inactivity, the World Health Organization (WHO) designed a global plan called Global Action Plan on Physical Activity 2018–2030 (GAPPA) in 2017. In this plan and according to the state of physical activity in 2016, actions and goals were set. However, the world is facing a COVID-19 pandemic, which has affected various aspects of lifestyle, including physical activity. Some studies have shown that physical activity reduced during the pandemic. For this reason, the WHO should review the GAPPA and update goals and actions according to the state of physical activity in 2020.

## Status of physical activity and GAPPA before the COVID-19 pandemic

It is well proven that regular physical activity can help to prevent and treat non-communicable diseases (NCDs) namely heart diseases, diabetes, and some cancers. It also helps to prevent risk factors of NCDs such as hypertension, overweight, and obesity and can improve quality of life, mental health, and well-being [1]. However and before the COVID-19 pandemic, global progress to increase physical activity has been slow. Worldwide, 1 in 4 adults, and 3 in 4 adolescents (aged 11–17 years), do not currently meet the physical activity global recommendations. The level of inactivity was about 70% in some countries, due to increased use of technology, changing patterns of transportation, and urbanization [1]. In addition to increasing the risk of many adverse health conditions, morbidity, and mortality, physical inactivity, and sedentary behavior pose a major economic burden worldwide [2]. Worldwide, the global economic cost of physical inactivity for the health care system was estimated at around $54 billion (INT$) in 2013 [3]. Besides, $13.7 billion productivity losses were due to physical-inactivity-related deaths [3].

In 2017, WHO endorsed a GAPPA (Global Action Plan on Physical Activity 2018–2030) to prevent and control inactivity and promote physical activity [1]. This plan was linked with the Sustainable Development Goals (SDGs) set for 2030. The target of the global action plan was a 10% and 15% relative reduction in the global prevalence of physical inactivity by 2025 and 2030, respectively (the baseline was 2016) [1]. However, Guthold et al. reported that with the pre-2018 trends, the 2025 global physical activity target will not be met [4].

## Physical activity status during COVID-19 pandemic

Although in some countries, various actions were carried out to reduce inactivity from 2018 to 2020, the COVID-19 pandemic affected the whole world in 2020. In many countries, governments have adopted restrictive measures such as quarantine, social distancing, the suspension of any social event, and the closure of schools, universities, gyms, sports centers, swimming pools, and public parks. All of these restrictions can reduce public access to physical activity opportunities.

Although in some countries, including Australia, pandemic presents an opportunity to introduce physical activity and its benefits and to consolidate new physical activity habits such as home-based exercise, many countries were affected by the pandemic. For example, Amini et al. examined the physical activity of the Iranian population during the COVID-19 pandemic and found that a total of 78% of the participants did not meet the physical activity guidelines during COVID-19 [5], while a previous study (before pandemic) has reported that about 33% of the adult population of Iran did not meet physical activity standard guidelines [4]. In Brazil, 79.4% of adults reported that their level of physical activity decreased during the COVID-19 pandemic [6]. Also, participants who felt a higher impact of quarantine on their physical activities tend to have a higher prevalence of depression and anxiety symptoms [6].

The closure of schools and universities during the COVID-19 pandemic has also increased inactivity. For example, Barkely et al. examined physical activity and sedentary behavior in a sample of university students and employees in pre and post the university canceled face-to-face classes and closed campus [7]. In this study, post-cancellation sedentary behavior was higher than pre-cancellation and physical activity was decreased from pre- to post-cancellation. Moreover, this decrease was greater in participants who were the most active pre-cancellation [7]. In children, Dunton et al. found that physical activity decreased during the COVID-19 pandemic so that this decrease was much more pronounced for older children (ages 9–13) vs. younger children (ages 5–8) [8].

Worldwide, Tison et al. reported a 5.5% (287 steps) and 27.3% (1432 steps) decrease in mean steps within 10 days and 30 days of the pandemic declaration, respectively [9]. These changes in average step count were different in countries. For example, in Italy, where the nationwide lockdown began on 9 March 2020, the reduction of step count was 48.7%. In contrast, in Sweden where has primarily advocated for social distancing and limitations on gatherings, this decrease was 6.9% [9]. Other studies also have reported decreased physical activity during the COVID-19 pandemic [10, 11].

## The necessity of updating the GAPPA

In general, many studies show that the COVID-19 pandemic has a negative effect on physical activity in some countries. Restrictions caused by pandemic encourage people to stay at home. Staying at home can also increase screen time and sedentary behavior. Due to the prolongation of the pandemic, this sedentary lifestyle can become a permanent lifestyle, which is especially problematic in children and adolescents. So, the WHO and other relevant organizations should consider ways to modify this lifestyle in the future.

Considering that the targets of the GAPPA have been set according to 2016 and considering the COVID-19 pandemic and its negative effects, it seems that new and more up-to-date targets are needed.

Some of the actions outlined in the GAPPA have been affected by the pandemic. In many countries, governments have adopted restrictive measures such as the closure of schools and universities. Therefore, strengthen the provision of good-quality physical education and more positive experiences and opportunities for active recreation, sports, and play for girls and boys in all pre-primary, primary, secondary, and tertiary educational institutions are missing. This loss of opportunity for physical activity in educational institutions, in particular, can harm physical literacy. Many behaviors and patterns of children and adolescents enter adulthood. Obese children and adolescents, for example, are more likely to have this obesity and its complications in adulthood. Therefore, special programs should be considered for this age group.

In many countries, many health care providers are involved in the COVID-19 and related programs. Therefore, they cannot have enough time to assess the patient and counsel on increasing physical activity and reducing sedentary behavior. Also, the number of people referring to primary and secondary health care centers has decreased due to fear of COVID-19.

Another action of GAPPA is to enhance the provision of, and opportunities for, more physical activity programs and promotion in parks and other natural environments. This measure is practically not feasible due to the closure of many of these centers. However, governments can make some centers available to the public only for walking and cycling, in accordance with protocols. About older adults, GAPPA recommends enhancing the provision of, and opportunities for, appropriately tailored programs and services aimed at increasing physical activity and reducing sedentary behavior, according to ability, in key settings such as local and community venues, health, social, and long-term care settings, assisted living facilities and family environments, to support healthy aging.

Older people are at high risk for the COVID-19, so they should not be encouraged to engage in physical activity outside the home. Also, since many older people need special exercises and sports movements (even in some of them, caregivers are needed to support them), prescribing exercises and physical activities online can be problematic.

In this period of the pandemic, some actions can be taken. One of these actions is to form campaigns. The WHO’s Be Active campaign aims to help people do just that—and to have some fun at the same time. The International Federation of Association Football (FIFA) has joined forces with the United Nations (UN) and the WHO in supporting the #BeActive campaign launched on the UN International Day of Sport for Development and Peace to encourage people to be #HealthyAtHome as the world comes together in the fight against COVID-19, today and every day. In some countries, national media campaigns have been formed. This approach is being implemented in some developed countries, including Australia and the UK. These countries have invested in national campaigns on TV, radio channels, prime-time, and social media to advocate for the importance of physical activity to maintain people’s health during the COVID-19 pandemic. The positive impact of such a media campaign has recently been observed in England. So, two thirds of adults reported consider exercise to be more important than ever during the current the COVID-19 pandemic [12]. Also, about 65% of people believed that exercise helped them maintain their mental health during the COVID-19 pandemic [12].

Also, mobile health apps may help promote active living during COVID-19. The positive effect of these apps on improving physical activity has been reported in some studies [13].

## Conclusion

COVID-19 was identified at the end of 2019 and has continued to this day. The end of it is still unknown. Therefore, it cannot be said that the effects of COVID-19 pandemic are short-term. This pandemic has promoted a sedentary lifestyle in all age groups. In general, it can be said that according to the existing conditions, there is a need to review and update the targets and actions of the GAPPA.

## Data Availability

Not applicable
